# Knowledge and attitudes toward human papillomavirus and cervical cancer prevention among women in the Asir Region, Saudi Arabia: a cross-sectional study

**DOI:** 10.3389/fonc.2025.1608568

**Published:** 2025-10-16

**Authors:** Geetha Kandasamy, Khalid Orayj, Eman Shorog, Asma M. Alshahrani, Tahani S. Alanazi, Hanan S. Algorman

**Affiliations:** ^1^ Department of Clinical Pharmacy, College of Pharmacy, King Khalid University, Abha, Saudi Arabia; ^2^ Department of Clinical Pharmacy, College of Pharmacy, Shaqra University, Dawadimi, Saudi Arabia

**Keywords:** cervical cancer, human papilloma virus, knowledge, attitude, women

## Abstract

**Background:**

Cervical cancer, mainly caused by Human Papillomavirus infection, is a growing public health concern in Saudi Arabia. Despite the availability of effective vaccination and screening programs in the country, awareness and acceptance remain limited, particularly among women in the Asir region. This study aimed to assess knowledge and attitudes toward Human Papillomavirus and cervical cancer prevention among women in the Asir region, Saudi Arabia.

**Methods:**

A cross-sectional study was conducted from January to March 2025 among women aged 18 and older in the Asir region of Saudi Arabia. A convenience sampling method was used to recruit participants. Data were collected through an online self-administered questionnaire, and multivariable logistic regression was performed to identify factors associated with knowledge and attitudes (p < 0.05).

**Results:**

Among the 523 participants, 66% demonstrated good knowledge of Human Papillomavirus and cervical cancer prevention, while 56% had positive attitudes. Awareness of Human Papillomavirus and the Human Papillomavirus vaccine was observed in 62.1% and 54% of women, respectively, while knowledge of screening tests remained limited at 32.7%. Only 21% of women reported being vaccinated against Human Papillomavirus. The main barriers discouraging women from practicing Pap tests were discomfort (65.4%) and lack of knowledge (63.7%). Factors significantly associated with lower odds of having good knowledge (Adjusted Odds Ratio, AOR) included older age (41–50 years: AOR = 0.479, 95% confidence interval (CI): 0.239–0.961; 51–60 years: AOR = 0.239, 95% CI: 0.113–0.505; >60 years: AOR = 0.127, 95% CI: 0.022–0.724), lower education (secondary school: AOR = 0.483, 95% CI: 0.305–0.764), and unmarried status (single: AOR = 0.562, 95% CI: 0.327–0.965; divorced/widowed: AOR = 0.413, 95% CI: 0.218–0.784). Negative attitudes were more prevalent among older women (41–50 years: AOR = 0.327, 95% CI: 0.170–0.629; 51–60 years: AOR = 0.272, 95% CI: 0.130–0.570; >60 years: AOR = 0.102, 95% CI: 0.012–0.876).

**Conclusion:**

The study demonstrated generally good knowledge and positive attitudes toward Human Papillomavirus vaccination and cervical cancer prevention among women in the Asir region; however, awareness of screening and vaccine uptake was suboptimal. Women who were older, less educated, or unmarried had lower knowledge and more negative attitudes. Targeted educational interventions through healthcare providers, school-based programs, and social media, along with improved healthcare access, are recommended to enhance awareness, encourage regular cervical cancer screening, and reduce disease burden.

## 1 Introduction

Cervical cancer (CC) is one of the most common gynecological cancers worldwide and a major public health concern, with over 600,000 new cases and 340,000 deaths reported globally in 2020, predominantly affecting women aged 30–49 years (1). Persistent infection with high-risk human papillomavirus (HPV), particularly types 16 and 18, accounts for the majority of cases worldwide (2). Despite the availability of effective vaccination and screening programs globally, awareness and uptake remain suboptimal in many regions. Globally, HPV vaccination and cervical cancer screening have shown substantial benefits in reducing incidence and mortality. However, knowledge gaps and limited uptake persist in many populations, especially among adolescents and young adults, who are ideal candidates for prophylactic vaccination (3, 4). Parental willingness to vaccinate children also significantly influences vaccine coverage (5–9). In Saudi Arabia, CC is the third most prevalent gynecological cancer, following ovarian and uterine cancers (10), and ranks as the ninth most common cancer among women, with an annual incidence of 358 new cases, predominantly affecting women aged 40–49 years (11). Persistent infection with HPV remains the primary cause, with types 16 and 18 accounting for 76% of cases both nationally and globally (11, 12). The age-standardized incidence rate (ASIR) of invasive cervical cancer among Saudi women rose from 1.23 per 100,000 in 2005–2009 to 2.45 per 100,000 in 2015–2019, with intermediate rates around 2.7 per 100,000 during 2010–2014 (13, 14). Although the age-standardized mortality rate (ASMR) remains low, continuous monitoring is warranted given the rising incidence.

This incidence is considerably lower than global averages (~14.1 per 100,000 women) but similar to neighboring Gulf countries such as Kuwait (4.8), the United Arab Emirates (5.4), and Qatar (6.8), where cervical cancer also ranks among the least common malignancies (15). However, despite this relatively low burden compared with international levels, the preventable nature of the disease and the rising trend in Saudi Arabia underscore the importance of strengthening preventive strategies. Nationwide studies highlight limited awareness and preventive behaviors. Between September and November 2023, cervical cancer screening prevalence was 22.1%, with 42.4% citing the lack of a physician’s recommendation as the primary barrier. Only 7.6% of participants had received the HPV vaccine, and over 84% demonstrated low knowledge about cervical cancer (16). Another survey across multiple regions found that while 84% of women were aware of cervical cancer, only 8% had ever undergone screening. Awareness of HPV transmission, genital warts, and its link to cervical cancer was below 20%, and only 2.0% had received the HPV vaccine despite 17.7% having heard about it (17).

The Asir region has distinctive demographic and healthcare characteristics compared to other parts of Saudi Arabia. Its largely rural and mountainous geography and uneven distribution of healthcare facilities limit access to specialized gynecological and preventive services compared to major urban centers such as Riyadh and Jeddah (18). Regional studies indicate low awareness and uptake of women’s health preventive measures in Asir, including cervical cancer and breast cancer screening (19, 20). Less than half of women in the Southern Region, which includes Asir, were aware of cervical cancer, and knowledge of HPV as a causal factor was particularly low (20). A recent survey in Asir also found substantial misconceptions about cervical cancer and limited awareness of HPV vaccination (21). These findings highlight regional disparities that may affect nationwide prevention programs, and this study provides the first comprehensive assessment of knowledge and attitudes toward HPV and cervical cancer prevention among women in Asir. In Saudi Arabia, cervical cancer screening is primarily opportunistic rather than population-based. The Ministry of Health recommends Pap smear and high-risk HPV testing for women aged 21–65, with screening intervals of every three years for those aged 21–29 and every five years for those aged 30–65 (22). The HPV vaccination schedule includes one- or two-dose regimens for girls aged 9–14 and young women aged 15–20, and two doses with a six-month interval for women over 21. The HPV vaccination program, initiated in 2008, targets girls aged 9–26 and boys aged 9–21 (22, 23).

The World Health Organization (WHO) recommends the HPV vaccine for females aged 9–14, as most individuals in this age group have not yet initiated sexual activity, and sexual contact is the primary mode of HPV transmission (24). The Advisory Committee on Immunization Practices (ACIP) recommends routine HPV vaccination for males at ages 11 and 12, alongside girls, with vaccination beginning as early as age 9. For older adolescents and young adults who were not adequately vaccinated at younger ages, the vaccine is recommended up to age 21 for males and up to age 26 for females (25, 26). In March 2022, the Saudi Ministry of Health incorporated the HPV vaccine into the Saudi Immunization Schedule through a school-based program targeting seventh-grade girls in collaboration with the Ministry of Education (27). Additionally, in September 2022, the vaccine became available for females aged 9–18 upon request through primary healthcare centers (27). Parental decision-making is crucial in adolescent vaccine uptake, influencing the success of these programs (28).

The Health Belief Model (HBM) is a widely used framework for understanding health behavior change and explains the underutilization of screening and preventive interventions for asymptomatic diseases. Its key constructs include perceived susceptibility, perceived severity, perceived benefits, perceived barriers, self-efficacy, and cues to action (29, 30). Educating women about vaccination benefits and addressing barriers is essential to enhance cervical cancer prevention. While several studies have examined HPV knowledge and attitudes in Saudi Arabia, data from the Asir region remain limited. Therefore, this study aimed to assess the knowledge and attitudes toward HPV and cervical cancer prevention among women in the Asir region. Understanding these factors is critical for designing effective public health interventions and policies to improve vaccine uptake and reduce the burden of cervical cancer. The findings will inform healthcare strategies aimed at increasing awareness and addressing barriers to HPV vaccination, supporting national efforts to reduce cervical cancer incidence.

## 2 Methods and materials

### 2.1 Study setting, design, and period

A cross-sectional study was conducted among women aged 18 years and older residing in the Asir region of Saudi Arabia from January to March 2025.

### 2.2 Sample size determination

The minimum required sample size was calculated using the Raosoft online sample size calculator (Raosoft Inc., Seattle, WA, USA) (31). Considering the estimated female population of 635,234 in the Asir region (General Authority for Statistics, 2022) (32), with a 95% confidence level, 5% margin of error, and an assumed response distribution of 50%, the required sample size was 384 participants. To improve the precision and reliability of the findings, recruitment continued beyond this minimum requirement. After applying the exclusion criteria (12 participants who did not provide consent and 11 with incomplete responses), the final sample analyzed included 523 participants.

### 2.3 Study population and sampling procedure

**Figure 1:**
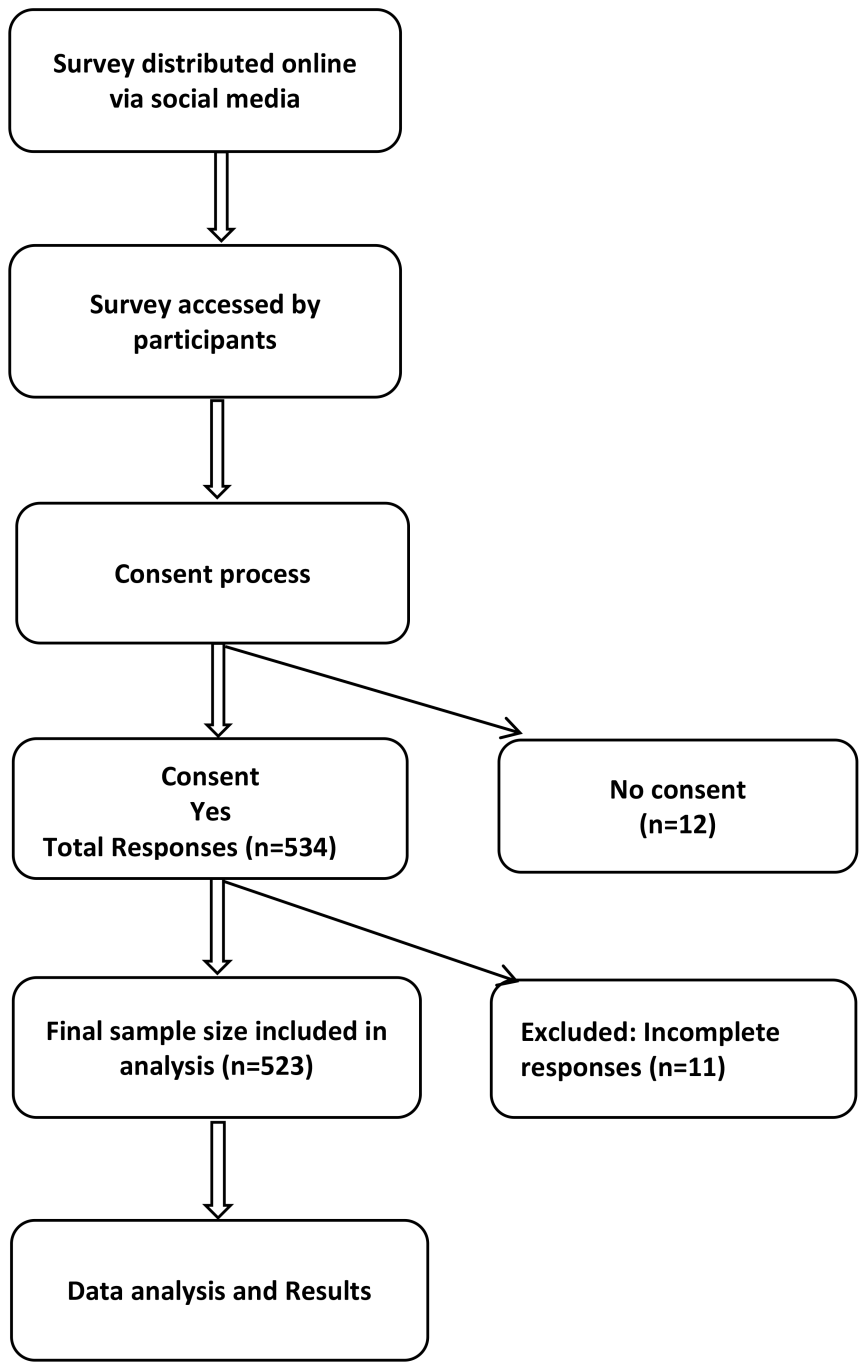
Flowchart of the sampling procedure among women in the asir region, Saudi Arabia (n=523).

Participants were eligible if they were women aged 18 years or older, residents of the Asir region, and willing to provide online consent. Women younger than 18 years, non-residents of the Asir region, or those who submitted incomplete survey responses were excluded from the study. Participants were recruited online using a convenience sampling method (**Figure 1**).

### 2.4 Data collection

Data were collected using an online self-reported questionnaire distributed via social media platforms, including X and WhatsApp (survey link: https://forms.gle/7vgFXaBNNq17p8VF6; accessed March 2, 2025). To accommodate the local population, the survey was made available in Arabic. Staff and students from King Khalid University assisted with distribution through targeted messages and posts, and outreach was also conducted at physical locations such as health clinics, universities, community centers, pharmacies, malls, parks, and religious centers using QR codes and posters to ensure broader participation. To minimize duplicate responses, the survey was configured to allow only one submission per device.

A standardized, self-administered questionnaire was developed to assess knowledge and attitudes regarding HPV, the HPV vaccine, and CC, based on previous literature (33–36). It was developed in English, translated into Arabic by bilingual experts, back-translated for accuracy, and reviewed by subject matter experts for cultural relevance and clarity. A pilot study with 50 participants assessed clarity and reliability, yielding a Cronbach’s alpha of 0.854, indicating good internal consistency. The questionnaire consisted of three sections. Section 1 collected sociodemographic data, including age, marital status, education (categorized according to the Saudi system: secondary school (grades 7–9), high school (grades 10–12), graduate (post-secondary or university), and uneducated), and employment status. Section 2: Knowledge was assessed using 10 scored questions and 2 qualitative items (Questions 11 and 12), which were not scored. Each correct answer was awarded 1 point, with a maximum possible knowledge score of 19. Questions 1–6, 9, and 10 were single-response items (yes/no or multiple-choice with one correct answer). Question 7 included 6 multiple-response items, and Question 8 included 5 multiple-response items, with each correct response receiving 1 point. Participants scoring ≥50% of the maximum were classified as having good knowledge, while those scoring <50% were classified as having poor knowledge.

Section 3 evaluated participants’ attitudes were evaluated using nine items. Questions 1–3 were single-response yes/no items, Questions 5–6 were single-response multiple-choice items (participants selected one option from several possible responses), and Questions 7–9 were single-response items with three options (Yes/No/Not sure). Each scored item was assigned 1 point for a response indicative of a positive attitude, yielding a maximum attitude score of 8. Question 4 was qualitative, assessing barriers to Pap test participation, and was not included in the scoring. Participants with scores ≥50% were classified as having a positive attitude, while those scoring <50% were classified as having a negative attitude. Question 3 assessed HPV vaccination history (for participants aged 9–26 years) and willingness to vaccinate daughters (restricted to participants with at least one daughter).

### 2.5 Variables

The study included both independent and dependent variables. The independent variables were demographic characteristics, including age, marital status, education level, and employment status. The dependent variables were participants’ knowledge and attitudes regarding HPV and cervical cancer prevention. Operational definitions: Women scoring ≥50% of the maximum were classified as having good knowledge or a positive attitude, while those scoring <50% were classified as having poor knowledge or a negative attitude, consistent with previous studies (37).

### 2.6 Statistical analysis

The survey responses collected through Google Forms were exported into Microsoft Excel and subsequently imported into SPSS for analysis. The data were cleaned, coded, and analyzed using SPSS version 18. Descriptive statistics were presented as frequencies and percentages for categorical variables. Multivariable logistic regression analysis was conducted to identify factors associated with knowledge and attitudes regarding HPV and CC prevention. Independent variables included demographic characteristics (age, employment status, education level, marital status), while dependent variables were categorized as good or poor knowledge and positive or negative attitudes. Adjusted odds ratios (AOR) with 95% confidence intervals (CI) were calculated to assess associations, and a p-value of <0.05 was considered statistically significant.

### 2.7 Ethical approval

Ethical approval was granted by the Institutional Review Board (IRB) of King Khalid University (ECM ≠2025-116). Participants were recruited online, and only those who provided consent were included. Confidentiality was emphasized throughout the recruitment process, and participation remained entirely voluntary. Upon clicking the survey link, participants were directed to the first page of the questionnaire on Google Forms, where they were presented with a consent form outlining the study’s objectives, voluntary participation, and confidentiality measures. Participants indicated their willingness to participate by selecting “Yes” or declined by selecting “No.” Those who declined were automatically redirected away from the questionnaire. Informed consent was therefore obtained online through this mandatory process. All data were securely stored in a password-protected database, accessible only to the principal investigator and authorized research team members, and anonymity was maintained by not collecting personally identifiable information.

## 3 Results

### 3.1 Demographics

Of the 523 women, the mean age was 34.7 ± 11.2 years, with a median of 32 years and an interquartile range (IQR) of 25–43 years. The majority were aged 18–30 years (222, 42.4%), followed by those aged 31–40 years (123, 23.5%) and 41–50 years (114, 21.8%). Participants aged 51–60 years were 56 (10.7%), while only 8 (1.5%) were above 60 years of age. Regarding employment status, 197 (37.7%) participants were employed, and 326 (62.3%) were unemployed. In terms of educational attainment, most had a graduate-level education 358 (68.5%), followed by 126 (24.1%) with secondary school education and 26 (4.9%) who completed high school. A small proportion, 13 (2.5%), were uneducated. As for marital status, 249 (47.6%) participants were single, 220 (42.1%) were married, and 54 (10.3%) were either divorced or widowed (**Table 1**).

**Table 1:** Sociodemographic characteristics of study participants in the Asir Region, Saudi Arabia (n = 523).

Variables	Category	Number (n) (%)
Age (Years)	Mean ± SD	34.7 ± 11.2
	Median (IQR)	32 (25–43)
	18-3	222 (42.4%)
	31-40	123 (23.5%)
	41- 50	114 (21.8%)
	51-60	56 (10.7%)
	Above 60	8 (1.5%)
Employment status	Employed	197 (37.7%)
	Unemployed	326 (62.3%)
Education level	Uneducated	13 (2.5%)
	Secondary school	126 (24.1%)
	High School	26 (4.9%)
	Graduate	358 (68.5%)
Marital status	Married	220 (42.1%)
	Single	249 (47.6%)
	Divorced/Widow	54 (10.3%)

### 3.2 Knowledge and attitudes

**Figure 2:**
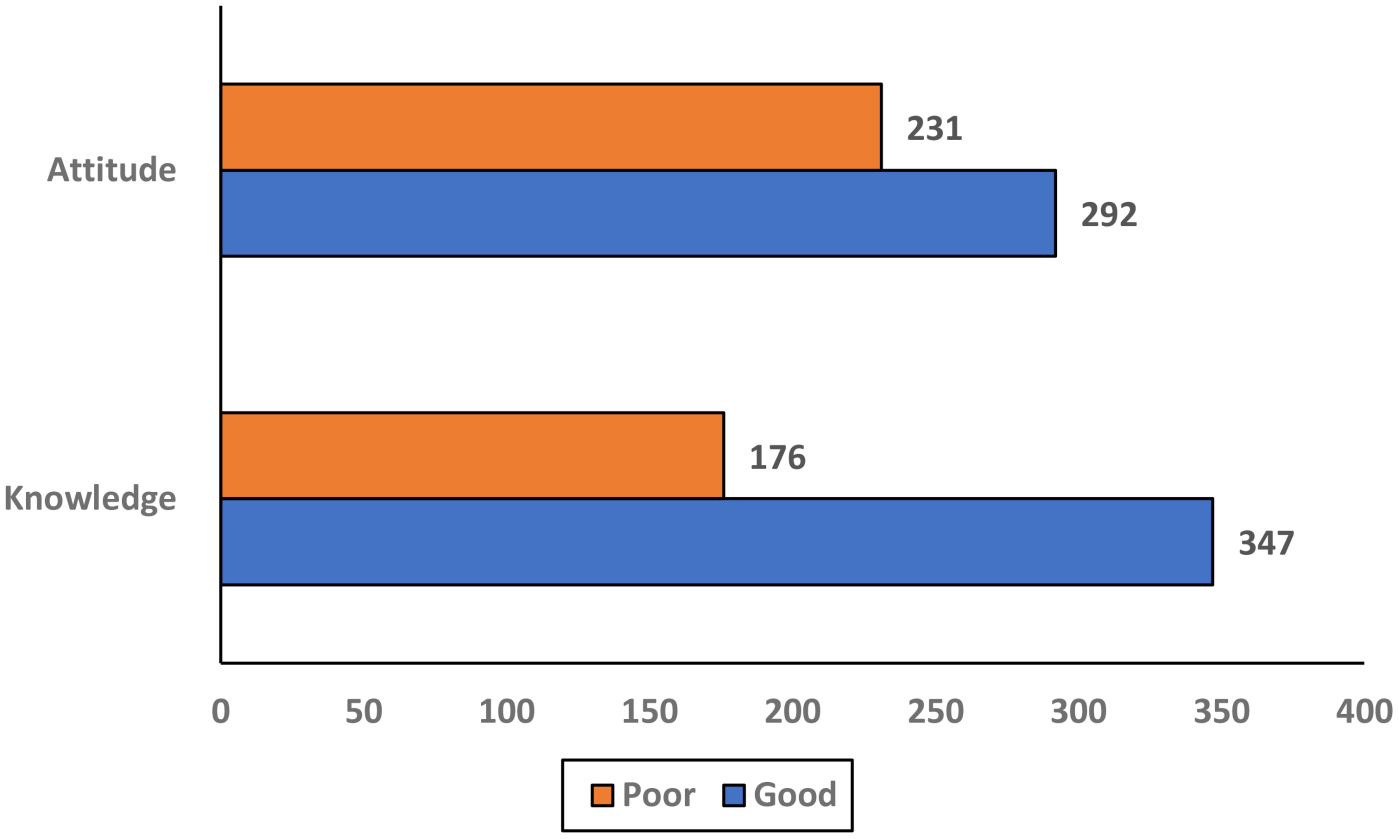
Scores of knowledge and attitudes toward human papillomavirus (HPV) and cervical cancer among women in the Asir Region, Saudi Arabia (n = 523).

**Figure 3:**
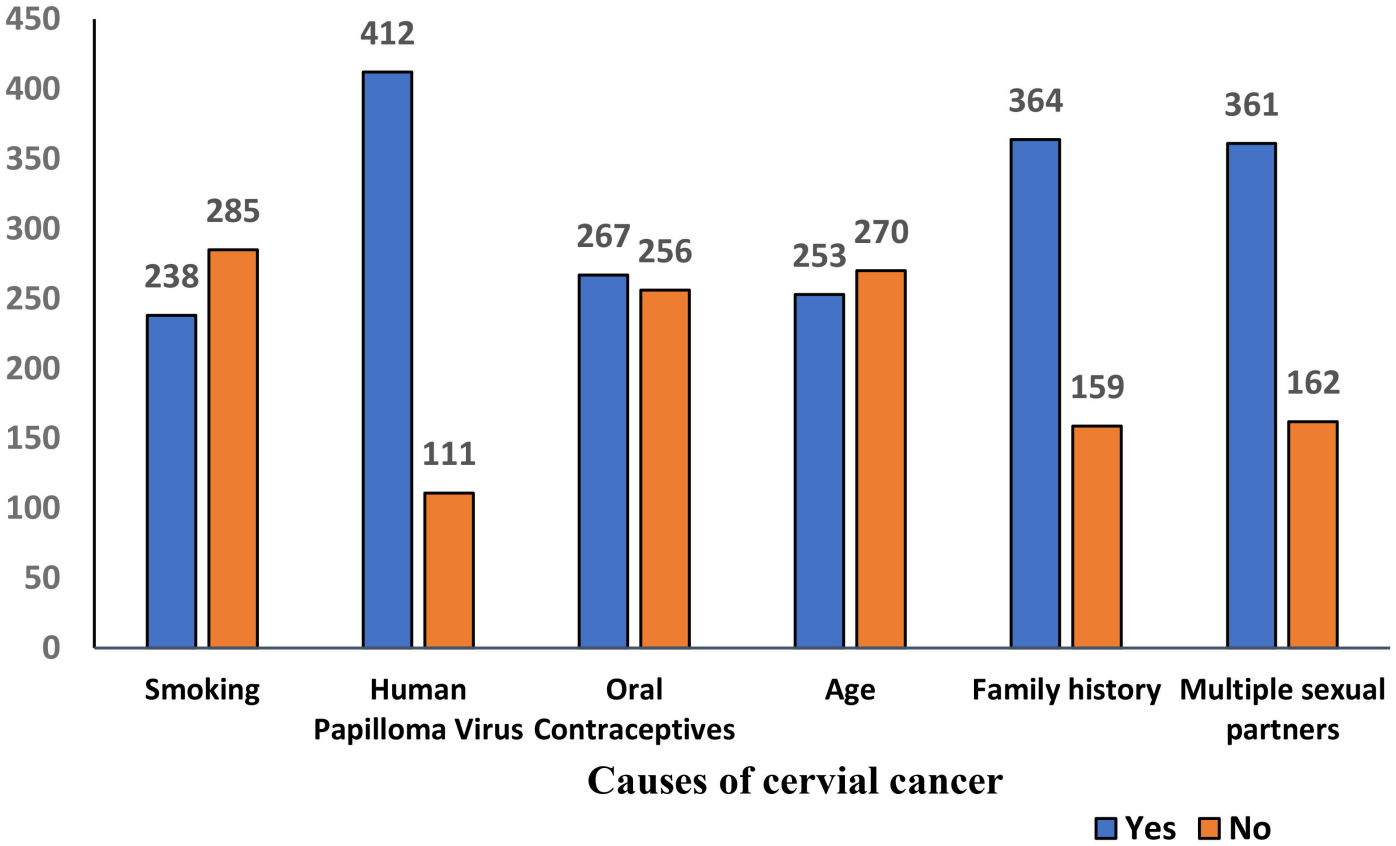
Causes of cervical cancer among women in the Asir Region, Saudi Arabia (n = 523).

**Figure 4:**
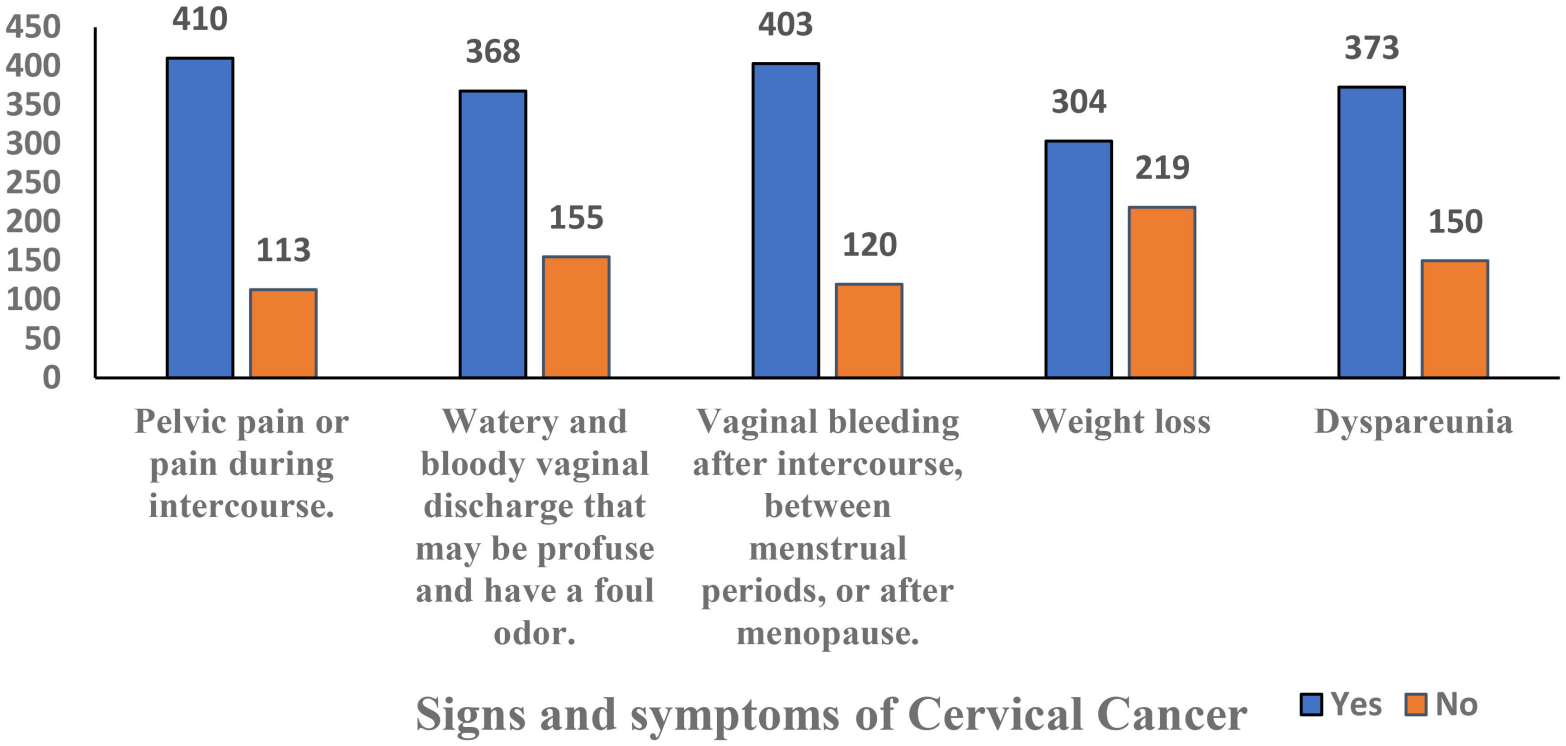
Signs and symptoms of cervical cancer prevention among women in the Asir Region, Saudi Arabia (n = 523).

Based on the predefined threshold of ≥50% of the maximum score (as described in the Methods), participants scoring ≥50% were classified as having good knowledge or a positive attitude, while those scoring below 50% were considered to have poor knowledge or a negative attitude. Good knowledge was demonstrated by 347 (66%) women, while 176 (34%) had poor knowledge. Positive attitudes were observed in 292 (56%) women, while 231 (44%) had negative attitudes (**Table 2**, **Figure 2**). Awareness of HPV was reported by 325 (62.1%) women, while 282 (54%) were aware of the HPV vaccine. Knowledge of screening tests for cervical cancer was limited, with only 171 (32.7%) aware of them. A majority 385 (68.5%) believed cervical cancer is preventable, while 138 (26.4%) disagreed. Awareness of the Pap smear test was reported by 241 (46.1%) women Regarding perceived causes of cervical cancer, 412 (78.8%) correctly identified HPV, while substantial proportions also reported family history 364 (69.6%) and multiple sexual partners 361 (69.0%) as risk factors. Other reported causes included smoking 238 (45.5%), oral contraceptives 267 (51.0%), and increasing age 253 (48.4%) (**Figure 3**). When asked about signs and symptoms of cervical cancer, most participants recognized pelvic pain or pain during intercourse 410 (78.4%), postcoital or intermenstrual vaginal bleeding 403 (77.1%), and foul-smelling watery or bloody vaginal discharge 368 (70.4%). Weight loss 304 (58.1%) and dyspareunia 373 (71.3%) were less frequently reported but still noted by more than half of the respondents. (**Table 3**, **Figure 4**). Regarding vaccination attitudes, 404 (77.2%) believed that women vaccinated against HPV still need a Pap test; however, only 110 (21%) were vaccinated against HPV. Among participants with at least one daughter (n=253), 180 (71.1%) expressed willingness to vaccinate their daughters, while 73 (28.9%) were not willing (**Table 4**).

**Table 2:** Scores of knowledge and attitudes toward human papillomavirus (HPV) and cervical cancer among women in the Asir Region, Saudi Arabia (n = 523).

Category	Knowledge n (%)	Attitude n (%)
Good	347 (66%)	292 (56%)
Poor	176 (34%)	231 (44%)

**Table 3:** Knowledge of human papillomavirus (HPV) and cervical cancer prevention among women in the Asir Region, Saudi Arabia (n = 523).

Questions	Yes n (%)	No n (%)
1. Are you aware of the virus responsible for causing cervical cancer?	232(44.4%)	291(55.6%)
2. Have you heard of Human Papillomavirus (HPV)?	325(62.1%)	198(37.9%)
3. Have you ever heard about a vaccine that protects against cervical cancer?	282(54%)	241(46.1%)
4. Do you know about the screening tests used to detect cervical cancer?	171(32.7%)	352(67.3%)
5. Is cervical cancer preventable, in your opinion?	385(73.6%)	138(26.3%)
6. Do you know what a Pap smear test is?	241(46.1%)	282(54%)
7. What causes cervical cancer? (Multiple responses)		
Smoking	238(45.5%)	285(54.5%)
Human Papilloma Virus	412(78.8%)	111((21.2%)
Oral Contraceptives	267(51%)	256(49%)
Age	253(48.4%)	270(51.6%)
Family history	364(69.6%)	159(30.4%)
Multiple sexual partners	361(69%)	162(31%)
8.What are the signs and symptoms of cervical cancer? (Multiple responses)		
Pelvic pain or pain during intercourse.	410(78.4%)	113(21.6%)
Watery and bloody vaginal discharge that may be profuse and have a foul odor.	368(70.4%)	155(29.6%)
Vaginal bleeding after intercourse, between menstrual periods, or after menopause.	403(77.1%)	120(22.9%)
Weight loss	304(58.1%)	219(41.9%)
Dyspareunia	373(71.3%)	150(28.7%)
9.When is the recommended time to have a Pap smear test performed? (Single correct answer)		
Every three years (From 30 to 65 years old)	114(28.8%)	
Every year (From 30 to 65 years old)	117(22.4%)	
Once in a life time	63(12%)	
I don’t know	229(43.8%)	
10.Do you think that HPV can get transmitted sexually (%)		
Yes	202(38.6%)	
No	231(44.2%)	
Don’t know	90(17.2%)	
11. The source of knowledge regarding the HPV (Choose anyone)		
Healthcare providers	159(30.4%)	
Through internet/social media	206(39.4%)	
Family members/Friends	64(12.2%)	
Television/radio/newspaper	37(7.1%)	
School studies/University studies	57(10.9%)	
12.Reasons for avoiding HPV vaccine (Multiple responses)		
Side effects	133(25.4%)	
Limited access to healthcare services	93(17.8%)	
Lack of knowledge	297(56.8%)	

**Table 4:** Attitudes regarding human papillomavirus (HPV) vaccination and cervical cancer prevention among women in the Asir Region, Saudi Arabia (n = 523).

Attitude Questions	Yes n (%)	No n (%)
1. Do women who have received the HPV vaccination still need to undergo a Pap test?	404(77.2%)	119(22.8%)
2. Have you ever been vaccinated against HPV?	110(21%)	413(79%)
3. Would you let your daughter receive the HPV vaccine when she is in school? (n = 253, only participants with at least one daughter)	180(71.1%)	73 (28.9%)
4. What might discourage you from getting Pap test practice? (Yes/no) (Multiple-response)		
Fear of discovering disease	294(56.2%)	229(43.2%)
Lack of knowledge and awareness	333(63.7%)	190(36.3%)
Lack of time	297(56.8%)	226(43.2%)
Cultural reasons	299(57.2%)	224(46.7%)
Way of examination	165(31.5%)	358(68.5%)
Discomfort	342(65.4%)	181(34.6%)
5. Who do you think can be vaccinated against human papillomavirus (HPV) and when should they get it? (Single-response)		
Only women over the age of 20.	90(16.9%)	
Women and men from 9 to 26 years old.	200(38.2%)	
Pregnant women.	45(8.6%)	
I don’t know.	188(35.9%)	
6. How important do you think it is for both men and women to be vaccinated against HPV? (Single-response)		
Very important	236(45.1%)	
Somewhat important	101(19.3%)	
Not important	56(10.7%)	
I don’t know	130(24.9%)	
7. Do you think that receiving the HPV vaccine can reduce the risk of developing cervical cancer? (Single-response)		
Yes	278(53.2%)	
No	58(11.1%)	
Not sure	187(35.8%)	
8. Do you believe that HPV Human Papillomavirus vaccination should be mandatory for all young people? (Single-response)		
Yes	92(17.6%)	
No	217(41.5%)	
I don’t know	214(40.9%)	
9. Do you feel comfortable discussing HPV vaccination with your healthcare provider? (Single-response)		
Yes	293 (56%)	
No	72 (13.8%)	
Not sure	158 (30.2%)	

### 3.3 Barriers and sources of information

**Figure 5:**
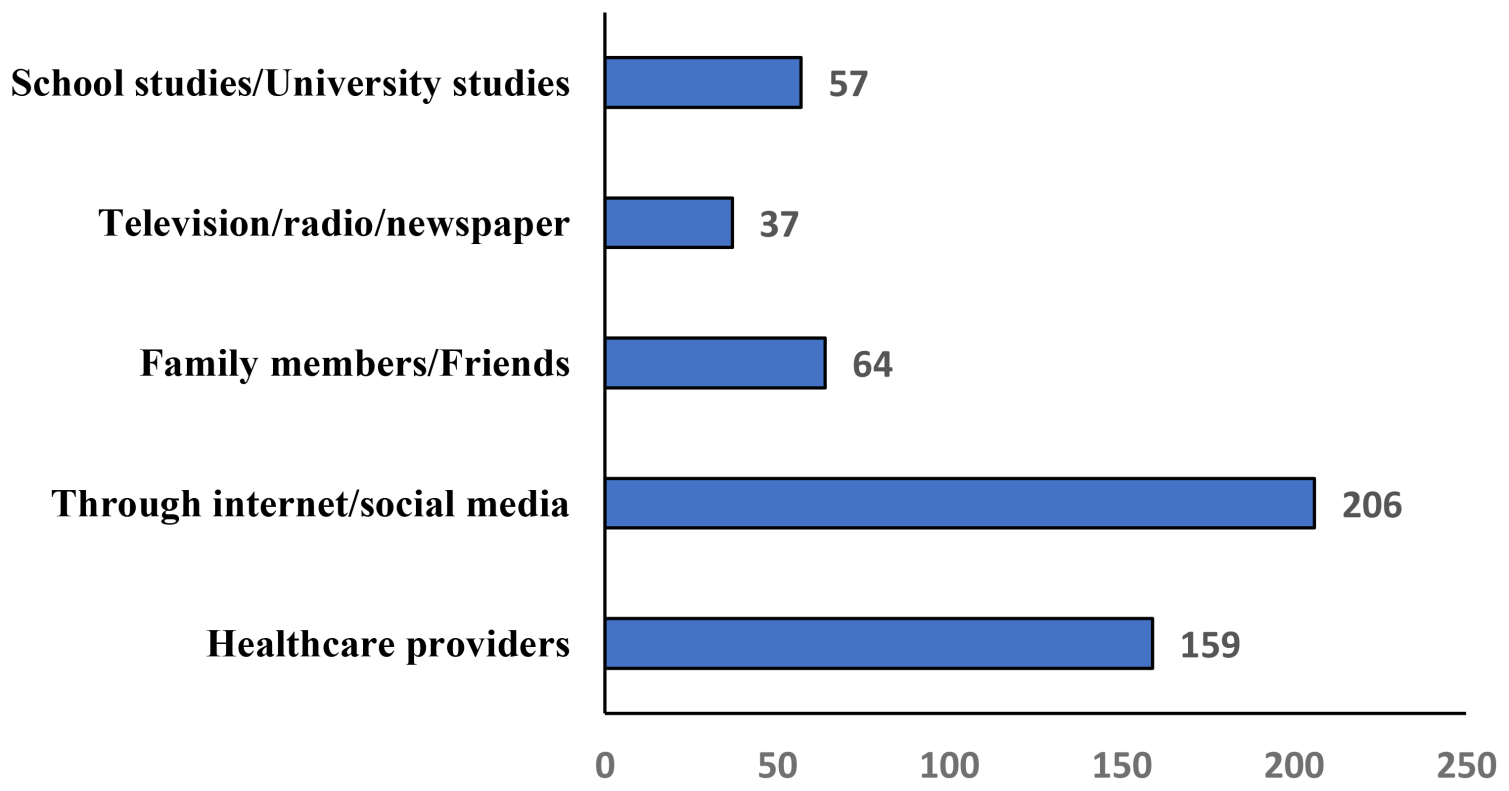
Sources of knowledge regarding human papillomavirus (HPV) and cervical cancer prevention among women in the Asir Region, Saudi Arabia (n = 523).

**Figure 6:**
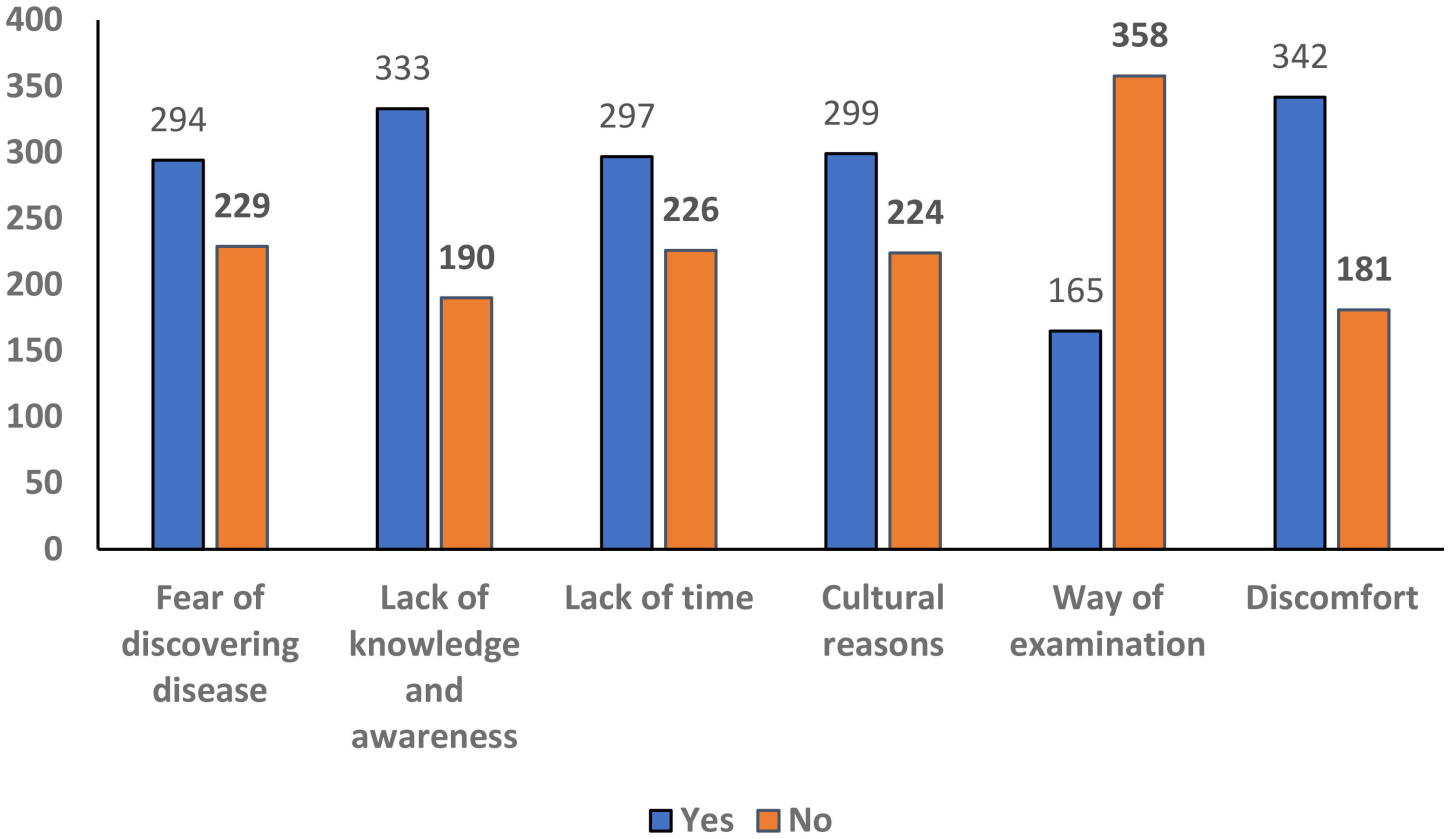
Factors discouraging women from undergoing Pap test in the Asir Region, Saudi Arabia (n = 523).

The most common sources of knowledge were the internet/social media 206 (39.4%), and healthcare providers 159 (30.4%) (**Table 3**, **Figure 5**). Discomfort 342 (65.4%) and lack of awareness 333 (63.7%) were the main barriers to Pap test practice (**Table 4**, **Figure 6**). In this study, discomfort was defined as both physical and emotional unease associated with undergoing a Pap test, encompassing physical pain, embarrassment, and anxiety related to the intimate nature of the procedure.

### 3.4 Regression analysis

The multivariable logistic regression analysis was performed to examine factors associated with knowledge and attitudes regarding HPV and cervical cancer prevention. Women aged 41–50 years had significantly lower knowledge (adjusted odds ratio (AOR) = 0.479, 95% confidence interval (CI): 0.239–0.961, p = 0.038), and those aged 51–60 years (AOR = 0.239, 95% CI: 0.113–0.505, p = 0.001) and above 60 years (AOR = 0.127, 95% CI: 0.022–0.724, p = 0.020) were even less likely to have adequate knowledge. Regarding education, women who had completed secondary school were less knowledgeable compared to graduates (AOR = 0.483, 95% CI: 0.305–0.764, p = 0.002), while other education levels and employment status were not significantly associated with knowledge. Marital status also influenced knowledge, with single women (AOR = 0.562, 95% CI: 0.327–0.965, p = 0.037) and divorced/widowed women (AOR = 0.413, 95% CI: 0.218–0.784, p = 0.007) showing lower knowledge than married women. For attitudes, older age was the only significant predictor of negative attitudes. Women aged 41–50 years had lower odds of positive attitudes (AOR = 0.327, 95% CI: 0.170–0.629, p = 0.001), with further decreases in the 51–60 years (AOR = 0.272, 95% CI: 0.130–0.570, p = 0.001) and above 60 years groups (AOR = 0.102, 95% CI: 0.012–0.876, p = 0.037). Employment, education, and marital status were not significantly associated with attitudes. Overall, older age, lower education, and being single or divorced/widowed were significantly associated with lower knowledge, while older age was the main factor associated with negative attitudes toward HPV and cervical cancer prevention (**Table 5**).

**Table 5:** Multivariable logistic regression of factors associated with knowledge and attitudes regarding HPV and cervical cancer among women in the Asir Region, Saudi Arabia (n = 523).

Independent variables	Groups	Knowledge			Attitude		
		Adjusted odds ratio (AOR)	95 % confidence interval (CI)	P value	Adjusted odds ratio (AOR)	95 % confidence interval (CI)	P value
Age	18–30 years	Reference					
	31–40 years	0.561	0.303-1.037	0.065	0.671	0.376-1.197	0.177
	41–50 years	0.479	0.239-0.961	0.038*	0.327	0.170-0.629	0.001*
	51–60 years	0.239	0.113-0.505	0.001*	0.272	0.130-0.570	0.001*
	Above 60 years	0.127	0.022-0.724	0.020*	0.102	0.012-0.876	0.037*
Employment status	Employed	Reference					
	Unemployed	0.994	0.611-1.616	0.981	0.535	0.333-0.859	0.242
Education level	Uneducated	0.318	-.091-1.108	0.072	0.536	0.145-1.977	0.256
	Secondary school	0.483	0.305-0.764	0.002*	0.769	0.490-1.207	0.253
	High School	1.069	0.418-2.733	0.889	1.165	0.479-2.836	0.596
	Graduate	Reference					
Marital status	Married	Reference					
	Single	0.562	0.327-0.965	0.037*	1.375	0.831-2.272	0.210
	Divorced/Widow	0.413	0.218-0.784	0.007*	0.710	0.372-1.357	0.301

*P<0.05 Significant.

## 4 Discussion

This study assessed the knowledge and attitudes of 523 women in Saudi Arabia regarding HPV and CC prevention. The findings showed that 66% of participants had a good level of understanding, while 34% demonstrated limited awareness. In terms of attitudes, 56% expressed a positive outlook, whereas 44% were less inclined to support preventive measures. These results underscore the need to strengthen public health initiatives to bridge knowledge gaps and promote HPV vaccination and CC screening. A significant portion of women in Saudi Arabia showed awareness of CC and HPV. Specifically, 44.4% of participants knew that HPV is responsible for causing CC, and 62.1% had heard of HPV. These findings align with previous research in Saudi Arabia (38, 39), which reported moderate levels of awareness. A prior study indicated that 60.85% of participants had heard about HPV, showing relatively similar trends (40). However, earlier studies highlighted poor understanding of CC (53.05%), HPV (51.79%), and the vaccine (34.09%) (40–42). Although awareness levels appear to be improving, many women still lack comprehensive knowledge about HPV and its link to CC, emphasizing the need for targeted educational interventions. Akkour et al. reported that 84.0% of respondents had heard about CC, and 61.7% of women acknowledged that early diagnosis leads to a better prognosis. However, only 18.1% knew that HPV causes CC, and just 21.1% understood the route of HPV transmission (40). In comparison, our study found that 44.4% of women were aware of the virus responsible for CC, indicating relatively higher awareness regarding the causal link between HPV and CC. This difference may reflect improvements in public health campaigns, greater availability of health information through digital platforms, and the younger age distribution of our participants (35, 41). The observed improvement in HPV awareness and vaccine uptake may reflect recent national health initiatives in Saudi Arabia, including school-based immunization programs and public health campaigns (43). Greater access to information via digital platforms and healthcare providers likely contributed to higher awareness, highlighting the need for sustained, culturally sensitive education to further enhance vaccination coverage, promote regular screening, and reduce cervical cancer burden (44).

Our study found that 73.6% of participants believed CC is preventable, while 26.3% disagreed. Knowledge of Pap smear testing remained limited, as only 46.1% were familiar with it, while 54% were unaware. Although 79.5% expressed a positive attitude toward screening, only 21.4% had undergone a Pap smear, revealing a gap between awareness and participation. Despite this high willingness, actual uptake remains low, reflecting a clear intention–behavior gap. This mismatch is primarily driven by lack of knowledge, cultural sensitivities, fear of diagnosis, and logistical constraints. Addressing these barriers through targeted educational campaigns, provider engagement, and accessible screening services is essential to convert willingness into action and improve cervical cancer prevention (45). National and regional data indicate low cervical cancer screening coverage in Saudi Arabia. Al Khudairi et al. (46) reported that 75.2% of women in Riyadh had never undergone a Pap smear, and 75.5% had never received a physician recommendation, with 82–93.9% unaware of appropriate screening timing. Similarly, Al Ghamdi found that only 21.1% of adult Saudi women had undergone the test (47). Low participation reflects gaps in awareness, physician engagement, cultural sensitivities, misconceptions about cervical cancer risk, fear of diagnosis, and geographic barriers, particularly in rural areas. The differences in Pap smear awareness and uptake between studies may also be due to participant characteristics such as urban vs. rural residence, educational level, and accessibility of healthcare facilities, as well as variations in study methodology (online surveys vs. in-person interviews) (48).

These findings align with previous studies in Saudi Arabia and the region. Alharbi et al. (49) reported good CC knowledge among women in Makkah, yet our study found that only 32.7% were aware of screening, and just 13.8% knew about Pap smears. Similarly, Al Khudairi et al. (50) found that nearly half of Saudi women were unaware of Pap smears, and over 75% had never undergone one. Alsous et al. assessed women aged 18–60 years across four Arabic-speaking countries (Jordan, Saudi Arabia, Egypt, and Lebanon) and reported poor HPV awareness, with better knowledge among younger, educated women, particularly healthcare professionals (51).

According to the present study, 21% of Saudi women had received the HPV vaccine, compared to 8.25% reported in another Saudi study (35). Similarly, previous study found that only 2.0% of adult Saudi women had been vaccinated against HPV (40), and Yang et al. reported comparable findings in adult populations (52). Khulud et al. reported an HPV vaccination rate of just 10.9% among adult Saudi women (53). These differences may be explained by variations in age distribution, regional contexts, timing of vaccination initiatives, cultural factors, and healthcare access. For instance, our study included women aged 18 years and above, with 42% aged 18–30, which may partly explain the relatively higher vaccination rate observed (54–56). A study conducted in Abha also found that sociodemographic factors significantly influenced HPV vaccine acceptance among young and adolescent females (56). Recent systematic reviews have shown that narrative-based education on the benefits of the HPV vaccine can enhance vaccination uptake, emphasizing the role of healthcare providers in promoting it (57, 58). Recent Saudi studies indicate that physicians’ knowledge and recommendations significantly influence HPV vaccine uptake (59), while parental and teacher attitudes affect schoolgirls’ vaccination decisions (60). Strengthening policy interventions and provider engagement is crucial for improving HPV vaccine coverage. Implementing culturally sensitive strategies can enhance vaccine acceptance and support CC prevention in Saudi Arabia. These findings reinforce the importance of targeted education, school-based programs, and active healthcare provider engagement to improve HPV awareness and vaccination coverage in Saudi Arabia. Similarly, Al-Naggar et al. (61) reported that young women’s perceptions and opinions significantly influenced their willingness to receive HPV vaccination, highlighting the importance of understanding cultural and personal beliefs when designing educational interventions). Strengthening policy interventions and provider engagement is crucial for improving HPV vaccine coverage. Implementing culturally sensitive strategies can enhance vaccine acceptance and support CC prevention in Saudi Arabia. In Saudi Arabia, HPV prevention faces challenges such as cultural sensitivities, low awareness, and misconceptions (62, 63). Despite the availability of screening, uptake remains low. Many women in this study reported discomfort (65.4%), lack of knowledge (63.7%), cultural concerns (57.2%), time constraints (56.8%), and fear of diagnosis (56.2%) as key barriers to Pap smear testing, while 31.5% avoided the exam itself. Addressing these barriers through culturally sensitive education and awareness campaigns is essential to improving screening uptake and HPV prevention (63, 64).

Only 38.2% of Saudi participants correctly identified that HPV vaccinations are available for both men and women aged 9 to 26, consistent with other studies showing low vaccine knowledge among adults (65). In contrast, 71% of respondents in a Canadian survey knew men could also be vaccinated (66). Enhancing access and awareness for both genders could improve vaccination uptake, particularly among males (67). Numerous health behavior theories emphasize the importance of awareness and knowledge as essential components for adopting health-protective behaviors (68). Previous research has shown a positive correlation between vaccine uptake and levels of HPV knowledge (69). Although the HPV vaccine is generally safe, mild side effects such as soreness, redness, swelling, fever, nausea, or muscle/joint pain may occur (70). In this study, 25.4% of women avoided vaccination due to concerns about side effects, aligning with Radwan et al., where 27.7% reported similar hesitations (71). Healthcare providers must proactively address misconceptions to strengthen confidence, improve acceptance, and enhance CC prevention.

Our study identified the internet and social media (39.4%) and healthcare providers (30.4%) as the primary sources of knowledge. Similarly, another study reported internet/social media (69%) as the main source of awareness, followed by healthcare workers (29.8%) (47). Turki et al. also found online platforms as the primary information source (72). In this study, only ~30% of women identified healthcare providers as their main source of HPV information. Prior Saudi studies have reported barriers such as time constraints, limited provider recommendations, discomfort discussing sexual health, and training gaps (59, 60). Addressing these challenges through provider education, routine counseling, and culturally sensitive communication could strengthen providers’ role as trusted sources (45). Additionally, 77% of respondents associated vaginal bleeding after intercourse, between menstrual cycles, or after menopause with CC, consistent with Zahid et al. (73) and Easwaran et al. (74). A study in India reported that 79.9% of participants were willing to vaccinate their daughters (75).

Overall, these findings highlight that older age, lower education, and being single or divorced/widowed were significant predictors of lower knowledge, while older age was associated with negative attitudes toward HPV and CC prevention. Targeted educational interventions, culturally sensitive awareness campaigns, healthcare provider engagement, and digital platforms can further enhance vaccination rates and CC screening uptake.

### 4.1 Limitations

This study has several limitations. First, its cross-sectional design limits the ability to establish causal relationships between knowledge, attitudes, and preventive practices related to HPV and CC. Additionally, the data relied on self-reported responses, which may be subject to recall and social desirability biases, potentially leading to inaccurate reporting of awareness and attitudes. This study also relied on an online self-administered survey, which may have led to overrepresentation of younger and more educated women. The cross-sectional study with online, social media-based recruitment strategy may have introduced selection bias, potentially underrepresenting women without internet access or those less active on social media, such as older, rural, or less-educated populations. Despite efforts to reach diverse participants through multiple channels including health clinics, community centers, pharmacies, malls, parks, and religious centers the sample may not fully represent the broader female population in the Asir region. Future studies should use probability-based or mixed-method sampling to improve generalizability.

Sampling bias may be present, as the study primarily included accessible and willing participants, and the convenience sampling method may limit the generalizability of the findings to the broader Saudi population. Moreover, the study’s regional focus may not fully capture cultural variations across the country. Furthermore, the study lacked qualitative insights to explore reasons behind low vaccination and screening rates and did not thoroughly examine potential confounding variables such as socioeconomic status and healthcare access. Lastly, the absence of longitudinal follow-up limits the ability to assess changes in knowledge, attitudes, and practices over time following awareness interventions.

## 5 Conclusion

This study highlights that while many women in the Asir region have good knowledge and positive attitudes toward HPV vaccination and preventive measures, awareness and participation in cervical cancer screening remain limited. Older, less-educated, and unmarried or divorced/widowed women showed gaps in knowledge and more negative attitudes, whereas younger, more educated, and married women were more knowledgeable and proactive. These findings emphasize the need for targeted educational interventions, enhanced healthcare access, and school-based vaccination programs. Leveraging healthcare providers and social media as communication channels can further strengthen preventive practices. Future research should focus on evaluating the effectiveness of such interventions and identifying additional barriers to HPV vaccination and screening uptake.

## Data Availability

The original contributions presented in the study are included in the article/supplementary material. Further inquiries can be directed to the corresponding author.
