# Correction: Knowledge and attitudes toward human papillomavirus and cervical cancer prevention among women in the Asir Region, Saudi Arabia: a cross-sectional study

**DOI:** 10.3389/fonc.2025.1726725

**Published:** 2025-10-27

**Authors:** Geetha Kandasamy, Khalid Orayj, Eman Shorog, Asma M. Alshahrani, Tahani S. Alanazi, Hanan S. Algorman

**Affiliations:** ^1^ Department of Clinical Pharmacy, College of Pharmacy, King Khalid University, Abha, Saudi Arabia; ^2^ Department of Clinical Pharmacy, College of Pharmacy, Shaqra University, Dawadimi, Saudi Arabia

**Keywords:** cervical cancer, human papilloma virus, knowledge, attitude, women

The title of this article was erroneously given as: “Knowledge and Attitudes Toward Human Papillomavirus and Cervical Cancer Prevention Among Women in the Asir Region, Saudi Women: A Cross-Sectional Study”.

The correct title of the article is “Knowledge and Attitudes Toward Human Papillomavirus and Cervical Cancer Prevention Among Women in the Asir Region, Saudi Arabia: A Cross-Sectional Study”.

The original version of this article has been updated.

